# The Mediating Role of Self-Efficacy in the Relationship Between Locus of Control and Resilience in Primary School Students

**DOI:** 10.3390/ejihpe15070138

**Published:** 2025-07-17

**Authors:** Asimenia Papoulidi, Katerina Maniadaki

**Affiliations:** Department of Social Work, University of West Attica, Egaleo, 12244 Athens, Greece; maniadaki@uniwa.gr

**Keywords:** resilience, self-efficacy, locus of control, primary school students, mediation

## Abstract

Resilience refers to an enduring and yet fluid characteristic that enhances children’s adaptation. It is a dynamic developmental process that is highly promoted by individuals’ internal characteristics, such as self-efficacy and locus of control. The present study examined whether self-efficacy mediates the relationship between locus of control and resilience among Greek primary school students. Participants were 690 students aged 9–12 years who were enrolled at primary schools in Greece in Grades 4, 5, and 6. Participants completed a questionnaire including measures assessing resilience, locus of control, and self-efficacy. Structural equation modeling using AMOS 26.0 was used to analyze the data. The results indicated that locus of control and self-efficacy function as significant predictors for all dimensions of resilience, while demographic characteristics such as gender and grade only predict some dimensions of resilience. The hypothesized model was a good fit to the data, and self-efficacy partially mediates the relationship between locus of control and resilience. Psychologists, instructors, and practitioners can develop and apply intervention programs in order to strengthen children’s resilience by enhancing their self-efficacy and helping them adopt an internal locus of control.

## 1. Introduction

During the last decades, the concept of resilience has received significant attention in the field of psychology due to the increasing frequency and severity of adversities and disasters taking place (Masten, 2021), such as the recent COVID-19 pandemic (Masten & Motti-Stefanidi, 2020). From a Developmental Systems Perspective, resilience refers to a system’s ability to effectively adjust and cope with challenges that endanger its functioning, survival, or future growth (Masten, 2019). Resilience involves the capacity to bounce back from difficult experiences, overcome obstacles, and maintain a sense of well-being and positive functioning in the face of adversity (Masten, 2001). It is not just the absence of distress or negative emotions, but also involves the ability to thrive and experience positive emotions, relationships, and experiences.

Resilience is recognized as a significant factor for lifelong health, well-being, and quality of life (Tang et al., 2022) and is highly affected by internal mechanisms and personality traits (Prince-Embury, 2006). Extensive research has shown that resilience can mitigate the negative effects of stressful events or traumatic experiences (Matheson et al., 2020), accelerate recovery in the face of adversity, and reduce the risk of developing mental health problems, such as depression, anxiety, and post-traumatic stress disorder (PTSD) (Konaszewski et al., 2021; Southwick & Charney, 2012). Age and gender have been found to influence resilience levels, with studies suggesting that older children may exhibit greater resilience due to more advanced cognitive and emotional regulation skills (Papakonstantinopoulou, 2018), while gender differences tend to vary depending on the specific dimensions of resilience assessed and the sociocultural context in which individuals develop (H. Chen et al., 2021; Sun & Stewart, 2007).

Resilience is a multidimensional construct (Liu et al., 2020; Masten, 2019) that encompasses a broad range of psychological, emotional, and social competencies, including self-awareness and empathy, as well as the presence of external sources of support, such as family and school. Research suggests that resilience is neither just a trait that the child has (nature) nor simply a product of the environment (nurture) (Kuldas & Foody, 2022). Rather, it is the dynamic interaction of internal and external resources with internal or external challenges (Schafer, 2022). Therefore, resilience can be viewed as a state of functioning that is influenced by a variety of individual characteristics, external supports, and stressors present at a particular time (Masten, 2007). From this point of view, resilience is a dynamic process, arising from the interplay between risk and protective factors (Höltge et al., 2021; Ungar, 2018). This understanding is further supported by the transactional social-ecological perspective, which conceptualizes resilience as a dynamic process that involves transactions between the individual and their ever-changing environment (Cefai, 2021; Kuldas & Foody, 2022; Wambua et al., 2024).

The literature has shown that locus of control and self-efficacy are two important individual factors that contribute to resilience. Rotter (1966) was the first to describe locus of control as the degree to which an individual believes that he/she has control over the events and outcomes of his/her life. People with an external locus of control believe that outcomes and events are determined by forces outside of their control, such as other people, fate and chance, whereas people with an internal locus of control believe that outcomes and events in their lives are primarily the result of their own actions, decisions, and efforts. Internal locus of control is associated with higher levels of well-being and adaptability and greater academic achievement (Gifford et al., 2006). Conversely, external locus of control is associated with lower educational attainment (Ng-Knight & Schoon, 2017), reduced participation in problem-solving activities (Cheng et al., 2013), and higher levels of anxiety and depression (Costantini et al., 2021). Even though locus of control refers to a personality trait and is considered to be relatively stable, there is evidence that there is a dynamic quality to the construct and it is modifiable with appropriate interventions (Jarrett et al., 2007).

Previous research has shown that internal locus of control is positively correlated with resilience in samples of adults (Çelik et al., 2015; Georgescu et al., 2019; Türk-Kurtça & Kocatürk, 2020) and adolescents (Cazan & Dumitrescu, 2017). Studies that have investigated adolescents whose parents are divorced have shown that those with an internal locus of control tended to have better resilience abilities (Felicia et al., 2022). Although the same relation between resilience and internal locus of control seems to be true for children as well (Werner & Smith, 1992), in-depth research in this area is still scarce (McGregor, 2018).

Another concept that is related to locus of control is self-efficacy (Schwarzer & Warner, 2013). Self-efficacy refers to a child’s belief in their ability to successfully complete tasks and achieve goals (Bandura, 1997). According to Bandura’s (1997) social cognitive theory, self-efficacy plays an important role in influencing one’s behavior. If a child has confidence in their ability to produce a desired outcome by controlling their own actions (“I can do it”), they are likely to be more self-motivated, take more initiative, and deal flexibly with difficult situations. Children with higher levels of self-efficacy are more likely to view challenges as opportunities for growth and to have a sense of control over their lives (Van Dinther et al., 2014). On the contrary, children with low self-efficacy often seem to avoid completing a school assignment, do not set high goals, and choose to engage only in simple educational tasks (Britner & Pajares, 2006). Self-efficacy is positively associated with resilience and is considered an important psychological resource that contributes to resilient functioning, especially in the face of challenges (Sagone & Indiana, 2017). Research on gender differences in self-efficacy presents mixed findings, with some studies indicating higher social self-efficacy in boys (D’Amico et al., 2013) and others showing higher general or academic self-efficacy in girls (Papakonstantinopoulou, 2018; Webb-Williams, 2014), while other studies report no significant gender differences (Kountrafouri et al., 2021), highlighting the need for further investigation across diverse contexts and age groups.

Previous studies have shown that self-efficacy can act as a mediator in various relationships where resilience as the outcome—for instance, between psychological well-being and resilience, between optimism and resilience (Sabouripour et al., 2021), between physical exercise and resilience (Jiang et al., 2025), as well as between self-esteem and resilience, and between problem-solving ability and resilience (Papakonstantinopoulou, 2018). These findings highlight the central role of self-efficacy in resilience development. According to Bandura’s social cognitive theory (1997), self-efficacy is a central psychological mechanism that shapes how individuals interpret and respond to challenges and is influenced by other psychological variables such as locus of control. Particularly, during the primary school years, locus of control may influence the development of self-efficacy. Children who perceive that they have control over the outcomes of their actions (internal locus of control) are more likely to develop confidence in their ability to manage specific challenges (self-efficacy). Supporting this pathway, Yiming et al. (2023) found that locus of control has a direct positive effect on adolescents’ self-efficacy, which in turn mediates the relationship between locus of control and adolescents’ physical activity.

This developmental sequence aligns with the transactional social-ecological model of resilience (Kuldas & Foody, 2022), which emphasizes the ongoing interactions between the individual and their environment. This framework is particularly relevant for understanding resilience in primary school children, as their cognitive, emotional, and social skills are still developing, and their beliefs about themselves and the world are highly influenced by family, school, and cultural context.

Most of the research in the areas mentioned above has primarily focused on adolescents and adults, often examining the interplay between various psychological variables and resilience. However, there is a notable gap in studies that directly explore the relationship among locus of control, self-efficacy, and resilience in younger populations, particularly primary school students. Addressing this gap, the present study aims to investigate the associations among locus of control, self-efficacy, and resilience in a sample of primary school students and contribute to the field by proposing a mediation model, in which self-efficacy serves as a mediator between locus of control and resilience.

This study’s main hypotheses are the following:

1. There are gender and grade differences in students’ levels of resilience, locus of control, and self-efficacy.
2. All subscales of resilience are positively related to self-efficacy and negatively related to external locus of control.
3. Self-efficacy and internal locus of control predict resilience.
4. Self-efficacy mediates the relationship between locus of control and resilience.

## 2. Materials and Methods

### 2.1. Participants

The sample consisted of 690 students, aged 9–12 years, who were enrolled at public primary schools in Greece in Grade 4 (*n* = 252), Grade 5 (*n* = 194), and Grade 6 (*n* = 244). Of the sample, 321 students were boys (46.5%) and 369 were girls (53.5%). Inclusion criteria required that participants were enrolled in Grades 4, 5, or 6 in a Greek public primary school, had parental consent to participate, and were proficient in Greek to ensure comprehension of the questionnaire items. Exclusion criteria included students with a diagnosed cognitive or developmental disorder and those with incomplete or invalid questionnaire responses, such as patterns of missing or inconsistent answers. The schools were selected from areas with different socioeconomic backgrounds in an attempt to increase the representation of the sample.

### 2.2. Operational Definitions of Variables

In this study, resilience is defined as a multidimensional construct encompassing internal strengths (e.g., self-awareness, empathy, goals) and perceptions of external support (from school, community, family, peers).

Self-efficacy is defined as students’ general belief in their ability to successfully perform tasks and handle challenges across different situations, independent of specific skills.

Locus of control refers to the extent to which students perceive life outcomes as being the result of their own actions (internal locus) versus external forces such as luck, fate, or other people (external locus).

### 2.3. Measures

#### 2.3.1. Resilience Youth Development Module

Resilience was measured by administering the Greek version of the Resilience Youth Development Module (RYDM; Nearchou et al., 2014). It is a self-report instrument, with 34 items rated on a 4-point Likert scale (1 = Not at all true to 4 = Very much true), that evaluates internal and external factors associated with resilience in primary school students. In particular, it assesses three internal characteristics (empathy, goals & aspirations, self-awareness/confidence) and four external sources of support (school, family, peers, and community). An example of an internal characteristic item is “I try to understand what other people go through”, while an example of an external characteristic item is “Outside of my home and school, there is an adult who really cares about me”. Each subscale yields a unique score. The Cronbach’s α coefficients for each subscale of resilience were as follows: 0.66 for Empathy, 0.57 for Goals and aspirations, 0.60 for Self-awareness, 0.78 for Community support, 0.74 for Peer support, 0.70 for School support, and 0.68 for Home support. Although the Cronbach’s alpha value for the Goals and Aspirations subscale was relatively low, it was retained due to its theoretical relevance and because other subscales demonstrated acceptable reliability.

#### 2.3.2. New General Self-Efficacy Scale (NGSE)

Self-efficacy was measured by administering the New General Self-Efficacy Scale (NGSE; G. Chen et al., 2001), an instrument that assesses general self-efficacy in children aged 9–12 years old, which was adapted in Greek by Alexopoulos and Asimakopoulou (2009). It contains 8 items rated on a 5-point Likert scale (1 = completely disagree to 5 = completely agree). An example item is “When facing difficult tasks, I am certain that I will accomplish them”. In the present study, Cronbach α was found to be 0.85.

#### 2.3.3. A Locus of Control Scale for Children

A locus of control scale for children (Nowicki & Strickland, 1973), adapted in Greek by Giannitsas (1988), was used to measure children’s internal and external locus of control. It is a self-report instrument with 20 items that are answered in a dichotomous Yes or No way. For instance: “Do you feel that one of the best ways to handle most problems is just not to think about them?”. It is a valid and reliable instrument which has been used in previous studies conducted with Greek students (Andreou, 2000; Georgiou et al., 2017). The internal consistency of the scale was assessed using the Kuder-Richardson Formula 20 (KR-20), appropriate for dichotomously scored items. The KR-20 coefficient for the current sample was 0.64.

### 2.4. Procedure

Ethics approval for this study was granted from the Ethics Research Committee of the University of West Attica (protocol number: 43965-05/05/2022)) and the Regional Directorate for Primary and Secondary Education in Attica (protocol number: 7700-5 May 2022). The researchers contacted the school principals in order to gain their approval and obtained informed consent from the parents, as well as verbal assent from the students who participated in this study. During regularly scheduled class hours, the researcher visited the schools and administered to children the self-report instruments.

### 2.5. Data Analysis

Descriptive statistics, Pearson correlations, and regression analysis were performed for the study variables using the IBM SPSS Statistics, Version 26. Structural equation modelling (SEM) was applied to examine whether the theoretical model fitted the data of the present study using the IBM AMOS Version 26 (IBM Corporation, Armonk, NY, USA).

To determine whether constructs were interpreted similarly across gender and grade levels, measurement invariance was assessed using multi-group confirmatory factor analysis (MGCFA). Configural, metric, and scalar models were tested sequentially. Following established guidelines (Brown, 2006), three fit indices were used to evaluate model fit at each level: Comparative Fit Index (*CFI* > 0.95), Root Mean Square Error of Approximation (*RMSEA* < 0.06), and Standardized Root Mean Square Residual (*SRMR* < 0.08). Measurement invariance was evaluated primarily through changes in *CFI* (Δ*CFI* < 0.01) as recommended by Cheung and Rensvold (2002).

The mediating effect of the variable was tested using a bootstrap estimation approach on 2000 samples and a percentile method corrected for 95% bias. In order to determine the model fitting adequacy, the following indices were used (Hooper et al., 2008): Comparative Fit Index (*CFI*), Tucker–Lewis Index (*TLI*), and Incremental Fit Index (IFI) with values greater than 0.90 indicating a good fit and with values around or greater than 0.95 indicating an excellent model fit, as well as Root Mean Square Error of Approximation (*RMSEA*) and Standardized Root Mean Square Residual (*SRMR*) with values less than 0.08 indicating a good model fit, while values less than 0.05 indicating an excellent model fit (Kline, 2011).

## 3. Results

### 3.1. Confirmatory Factor Analyses

Confirmatory factor analyses (CFAs) were conducted on the following scales: Resilience Youth Development Module, New General Self-Efficacy Scale, and Locus of control scale for children. As indicated by Nearchou et al. (2014), the CFAs were applied separately for the internal (Empathy, Goals and Aspirations, and Self-awareness) and the external assets (School Support, Home Support, Peer Support, and Community Support) of the RYDM. For the internal assets, the model demonstrated an acceptable fit to the data: χ^2^(62) = 198.04, *p* < 0.001, *CMIN/DF* = 3.19, *CFI* = 0.868, *TLI* = 0.834, *GFI* = 0.957, *AGFI* = 0.937, and *RMSEA* = 0.056 [*LO* = 0.048, *HI* = 0.065]). Although the *CFI* and *TLI* values were slightly below the conventional threshold of 0.90, the *RMSEA* and absolute fit indices indicated an adequate to good overall model fit. For the external assets, the model demonstrated an acceptable fit to the data: χ^2^(183) = 569.52, *p* < 0.001, *CMIN/DF* = 3.11, *CFI* = 0.892, *TLI* = 0.876, *GFI* = 0.925, *AGFI* = 0.905, and *RMSEA* = 0.055 [*LO* = 0.050, *HI* = 0.061]). Although the χ^2^ test was significant—likely due to sample size—the *RMSEA* and other indices indicated a satisfactory model fit.

The New General Self-Efficacy Scale demonstrated an acceptable fit to the data: χ^2^(20) = 75.73, *p* < 0.001; *CMIN/DF* = 3.79; *GFI* = 0.972; *AGFI* = 0.949; *CFI* = 0.961; *TLI* = 0.946; *RMSEA* = 0.064 [*LO* = 0.049, *HI* = 0.079]); and *RMR* = 0.027. The results support the unidimensional structure of self-efficacy and suggest that the measurement model fits the data well. The locus of control scale for children demonstrated acceptable overall fit: χ^2^(135) = 316.38, *p* < 0.001, *CMIN/DF* = 2.34, *CFI* = 0.771, *TLI* = 0.740, *GFI* = 0.950, *AGFI* = 0.936, *RMR* = 0.011, and *RMSEA* = 0.044 [*LO* = 0.038, *HI* = 0.050]. While the comparative fit indices (*CFI*, *TLI*) fell below recommended thresholds, the model showed acceptable overall fit, particularly evidenced by strong absolute fit indicators such as *RMSEA* and *GFI*. The Hoelter index was 356 at the 0.05 level, indicating adequate sample size and model stability.

### 3.2. Descriptive Statistics

Mean scores and standard deviations for all the subscales of resilience, as well as the variables of self-efficacy and locus of control for each gender, each grade, are presented in Table 1. It was noticed that the mean score for the subscale of goals and aspirations was 14.49 (SD = 1.76), which is relatively high within its possible range (4–16). For the locus of control variable, the mean score was 6.49 (SD = 3.38), within a range of 0–20.

### 3.3. Measurement Invariance Across Gender and Grade

Based on Δ*CFI* values, scalar invariance across gender was supported for the Self-Efficacy scale and the External Factors subscale, indicating that comparisons of latent means between boys and girls are valid for these constructs. The Internal Factors subscale demonstrated only metric invariance, and the Locus of Control scale failed to meet scalar invariance, thus limiting the interpretability of observed group differences for these constructs (Table 2).

Across grade levels (4th–6th), scalar invariance was supported for the Self-Efficacy scale, indicating equivalence of measurement across age groups. Partial support was found for the External Factors subscale (metric but not scalar invariance). In contrast, scalar invariance was not established for the Internal Factors subscale and the Locus of Control scale, as Δ*CFI* exceeded acceptable thresholds (Table 3). This limits the validity of cross-grade comparisons of latent means for these constructs and suggests that observed group differences should be interpreted with caution.

### 3.4. Group Differences

Independent samples t-tests were conducted to examine gender differences across the study variables. However, scalar invariance across gender was not established for all constructs, indicating that direct comparisons of latent means may not be fully interpretable. Despite this limitation, descriptive differences were observed: girls scored significantly higher than boys in Empathy [*t*(688) = −2.76, *p* = 0.006] and Peer Support [*t*(634.994) = −3.94, *p* < 0.001], based on the adjusted α = 0.0071 (Bonferroni correction). No significant differences were found in Self-awareness, Home Support, or Self-Efficacy under the corrected threshold, although boys showed higher means on those variables.

A one-way ANOVA was performed to explore differences across grade levels (4th–6th). Scalar invariance was confirmed only for the Self-Efficacy scale, supporting valid mean comparisons for that construct. For other variables, such as Locus of Control and the Resilience subscales, scalar invariance was not supported, limiting interpretation. Notwithstanding, statistically significant mean differences emerged in Self-awareness [*F*(2, 687) = 6.62, *p* = 0.001], School Support [*F*(2, 687) = 3.46, *p* = 0.032], Self-efficacy [*F*(2, 687) = 4.61, *p* = 0.010], and Locus of Control [*F*(2, 687) = 3.23, *p* = 0.040]. Post-hoc Tukey HSD comparisons indicated that fourth graders scored significantly higher than sixth graders, while fifth graders did not differ significantly from either group.

### 3.5. Correlations Among Study Variables

Table 4 presents the correlation matrix based on observed scale scores, with significant correlations being evident among all variables. The strongest correlations were found between self-efficacy and self-awareness (*r* = 0.496, *p* < 0.001), empathy and self-awareness (*r* = 0.407, *p* < 0.001), as well as school support and home support (*r* = 0.449, *p* < 0.001). External Locus of control was statistically significantly negatively correlated with all the subscales of resilience and self-efficacy.

Summary: The correlation analysis revealed meaningful relationships among the study variables. Notably, self-efficacy showed a strong positive association with self-awareness, suggesting that students with greater confidence in their abilities are also more aware of their internal states and behaviors. The strong correlation between school and home support highlights the complementary role of different environments in fostering resilience. The consistent negative correlations between external locus of control and all resilience subscales underscore the detrimental impact of perceiving outcomes as externally controlled on children’s adaptive capacities and self-beliefs.

### 3.6. Regression Analysis

A stepwise regression analysis was conducted to determine which factors predict each of the subscales of resilience. The stepwise regression included the following predictor variables: gender, class, self-efficacy, and locus of control. As it is depicted in Table 5, empathy was predicted by gender, with girls having higher scores than boys, self-efficacy, and locus of control. Goals and aspirations were predicted by self-efficacy and locus of control. Self-awareness was predicted by grade, with lower grades having higher scores than upper grades, self-efficacy, and locus of control. Community support was predicted by self-efficacy and locus of control. Peer support was predicted by gender, with girls having higher scores than boys, self-efficacy, and locus of control. School support was predicted by gender, with girls having higher scores than boys, self-efficacy, and locus of control.

Summary: The stepwise regression analyses indicate that both self-efficacy and locus of control consistently emerged as significant predictors across all resilience subscales, highlighting their central role in shaping different dimensions of resilience. Gender was a significant predictor of empathy, peer support, and school support, with girls scoring higher than boys in these domains, suggesting potential gender-related differences in emotional and social aspects of resilience. Grade level predicted self-awareness, with younger students reporting higher levels than older students, possibly reflecting developmental shifts in self-perception. Overall, the findings underscore the importance of internal psychological resources (self-efficacy, locus of control) alongside demographic variables in explaining children’s resilience-related capacities.

### 3.7. Mediation Analysis

**Figure 1:**
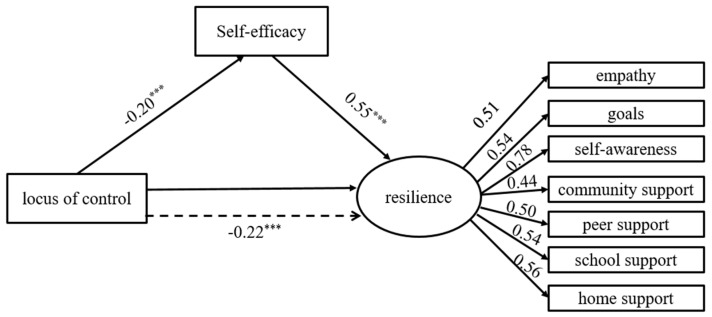
Mediation analysis. The dashed arrow between “Locus of Control” and “Resilience” represents an indirect effect, while solid arrows indicate direct effects. The triple asterisk symbol (***) denotes statistical significance at *p* < 0.001.

Mediation analysis was performed to assess the mediating role of self-efficacy in the relationship between locus of control and resilience. Overall, the SEM results revealed that the hypothesized model was a good fit to the data (*RMSEA* = 0.04, *SRMR* = 0.02, *GFI* = 0.98, *AGFI* = 0.97, and *CFI* = 0.98). The results revealed a significant direct effect of locus of control on resilience (β = −0.217, *p* = 0.001), a significant direct effect of locus of control on self-efficacy (*β* = −0.196, *p* = 0.001) as well as a significant direct effect of self-efficacy on resilience (*β* = 0.552, *p* = 0.001). The indirect effect of locus of control on resilience was again significant (*β* = −0.108, *p* = 0.001). This shows that self-efficacy partially mediates the relationship between locus of control and resilience (Figure 1).

Summary: These findings suggest that self-efficacy may function as a proximal psychological mechanism through which locus of control influences resilience. In line with Bandura’s social cognitive theory, self-efficacy reflects children’s belief in their ability to manage specific challenges, and this belief may be shaped by more generalized control beliefs such as locus of control. Thus, children who perceive greater personal control over life events may develop stronger self-efficacy, which in turn supports more resilient functioning.

## 4. Discussion

Although the literature about the factors that are involved in developing resilience is rich, personality traits are among the most important (Fayombo, 2010). Self-efficacy and internal locus of control are personal protective factors that are highly correlated with resilience. Previous studies with adolescents (Cazan & Dumitrescu, 2017) have shown a negative relation between external locus of control and resilience. A negative correlation between external locus of control and self-efficacy is also found in the present study, as well as in other studies with university students (Thompson et al., 2020) and high-school students (Anderson et al., 2005). Also, self-efficacy is positively related to all the subscales of resilience and particularly to self-awareness, a result which is also found in other studies with Greek students (Dimakos & Papakonstantinopoulou, 2015). Although few studies have tested general self-efficacy in children below the age of 12 (Schwarzer & Warner, 2013), it is a very important personality trait that characterizes those children who remain resilient despite adversity (Hamill, 2003). People with high self-efficacy and a high internal locus of control believe they have control over future events and use that control to achieve a positive result and experience enhanced well-being. As regards the subscales of resilience, the strongest correlations were found between empathy and self-awareness, as well as school support and home support, a finding which is also confirmed by Papazis (2020). Receiving support from school and family is considered a significant factor for children’s and adolescents’ life satisfaction and their psychological well-being (Tsouvelas et al., 2022).

In relation to gender differences, the present study showed that differences between boys and girls were found for empathy and peer support, in which girls scored higher. These findings align with the study by Nearchou et al. (2014), which examined Greek elementary school students’ resilience, as well as the study by Papazis (2020) in a sample of Greek adolescents. Higher scores for girls in the subscale of empathy were also found by Giannopoulou (2020) in a sample of 5th and 6th-grade Greek students. There seems to be a consensus in the literature as regards the subscales of resilience for which gender differences exist. These patterns may reflect gendered socialization processes that shape how boys and girls experience and express resilience-related competencies. For instance, girls may be more attuned to interpersonal dynamics and emotional expression due to social expectations around caregiving and relational sensitivity, which could explain their elevated scores in empathy and peer support. Such differences underscore the importance of considering gender-specific pathways in the design of resilience-enhancing interventions and call for theoretical models that account for the interplay between individual, social, and cultural influences on resilience development (Sölva et al., 2023).

As regards the concept of self-efficacy, findings on gender differences remain inconsistent. D’Amico et al. (2013) found that boys exhibited a higher perception of social self-efficacy compared to girls, while other studies have shown that girls report higher general or academic self-efficacy than boys (Dimakos & Papakonstantinopoulou, 2015; Webb-Williams, 2014). There are also studies reporting no statistically significant gender differences (Kountrafouri et al., 2021), which aligns with the findings of the present study. These inconsistencies may partly stem from methodological variations, including the types of self-efficacy measured (e.g., social vs. academic), the psychometric tools employed, and contextual or cultural differences across studies. Divergent operational definitions of self-efficacy—whether viewed as a domain-specific or general belief—may further complicate comparisons. Also, these differences might be attributed to situational factors such as classroom climate, teacher expectations, and social norms regarding gender roles. Therefore, it is important to examine the factors that influence how boys and girls perceive their abilities, as these can shape self-efficacy beliefs in ways that may differ across various educational and cultural contexts.

For the variable of locus of control, the present study did not find differences between boys and girls, a finding which is also confirmed by Sherman (1984) and Feingold (1994). On the contrary, Manger and Eikeland (2000) showed that 14- and 15-year-old girls had significantly higher internal locus of control scores than boys. Any differences in locus of control could be explained in terms of boys’ and girls’ general socialization experiences. From early childhood, boys and girls are exposed to different societal expectations and cultural norms that influence their beliefs about personal control and agency. For instance, girls are frequently encouraged to develop autonomy, self-regulation, and problem-solving skills, whereas boys are often socialized to be more risk-taking, competitive, and externally focused (Carter, 2014; Leaper & Friedman, 2007). Parenting styles and educational practices may also reinforce these differences (Krishna et al., 2024).

In examining the differences across the three grades, the present study found that fourth graders scored higher on the variables of Self-awareness, School Support, Self-efficacy, and Locus of Control. This finding is not entirely inconsistent with previous research. For instance, Nearchou et al. (2014) found differences only for the Goals and Aspirations subscale, with sixth graders outperforming fifth graders, while Giannopoulou (2020) found differences only for the subscale of empathy and community support. Although some studies support that children’s resilience increases with age (Papakonstantinopoulou, 2018), others report no significant influence of age on resilience (Gomez-Baya et al., 2020; Hunter, 2001). Regarding self-efficacy, the finding of the present study that fourth graders reported higher levels than sixth graders was in line with the study by Papakonstantinopoulou (2018) who found that fifth graders showed more self-efficacy that sixth graders but contrasts with the study by Kountrafouri et al. (2021), which found no statistical significant differences across grades. These results may reflect developmental and contextual dynamics. As students progress through school, they may face increasing academic pressure, social expectations, and transitional stressors, which could negatively affect their perceived resilience and self-efficacy. Additionally, this trend could be influenced by differences in self-perception and cognitive maturity across age groups. Such variations underline the importance of considering developmental stage and school-related stressors when assessing resilience in educational settings.

As regards locus of control, results seem to be more homogeneous. The finding of the present study that younger children have higher scores in external locus of control than older children is also confirmed by the longitudinal analysis conducted by Sherman (1984). Also, Nowicki and Strickland (1973) have supported that students’ responses become more internal with age. This developmental trend may reflect the gradual maturation of cognitive and metacognitive skills, which allow older children to better understand the relationship between their actions and outcomes.

Moreover, results support both self-efficacy and locus of control as significant predictors for all dimensions of resilience. These factors explained 27% of the variation in self-awareness and 12% of the variation in goals and aspirations, school support, and family support. Gender only predicted the dimension of empathy and peer support, while grade predicted only the dimension of self-awareness. The findings are in line with some other studies (Hamill, 2003; Papakonstantinopoulou, 2018), according to which resilience can be largely predicted by the person’s self-efficacy. Also, previous research by Leontopoulou (2006) has shown that locus of control operates as a significant predictive factor for resilience in adolescence.

In the present study, self-efficacy was found to partially mediate the relationship between locus of control and resilience, suggesting that general control beliefs may influence children’s confidence in managing challenges, which in turn supports resilient functioning. Other studies have explored the mediating role of self-efficacy in the relationship between psychological well-being and resilience, as well as optimism and resilience (Sabouripour et al., 2021). Most of the existing literature supports the view that self-efficacy is a predictor rather than an outcome of resilience (Jiang et al., 2025). In the Greek context, Papakonstantinopoulou (2018) has found that self-efficacy was the most important mediating factor in the relationship between self-esteem and resilience, as well as between problem-solving ability and resilience. These findings align with Bandura’s (1997) social cognitive theory, according to which self-efficacy plays a central role in human agency, influencing how individuals respond to challenges and stress. High self-efficacy enhances motivation, persistence, and adaptive coping strategies, all of which are essential components of resilience. From this perspective, self-efficacy serves as a foundational mechanism that enables individuals to effectively manage adversity and develop resilient outcomes.

## 5. Limitations and Future Research

Some limitations of this study should be addressed. One limitation regards its cross-sectional nature. Data were collected at one time-point of the school year and therefore lack temporal depth. The cross-sectional design limits the ability to capture resilience as a developmental process over time and prevents conclusions about causality. A longitudinal data-collection design would allow for a deeper exploration of developmental pathways, help confirm the stability of relationships between variables over time, and provide valuable insights into long-term resilience processes. Second, data collection was implemented using self-report measures. Although assessing resilience and personal traits, such as self-efficacy and locus of control through self-report instruments is a common, widely used and reliable method, future studies could try to collect data from multiple sources (i.e., parents, teachers) in order to verify the results and avoid any possible type of bias in reporting (i.e., social desirability bias).

Another limitation of the present study concerns the partial support for measurement invariance across gender and grade levels. While scalar invariance was confirmed for certain measures (e.g., Self-Efficacy), it was not established for others, such as the Internal Factors subscale and the Locus of Control scale. This restricts the interpretability of group comparisons for these constructs, as observed mean differences may reflect measurement bias rather than true differences. Future research should further examine the cross-group equivalence of these instruments and consider the use of alternative scales or refined items to ensure valid comparisons. Finally, the current study investigated only the personal protective factors of self-efficacy and locus of control and did not include measures of contextual factors, which limits the generalizability of the findings. It is important to acknowledge that cultural norms, teacher and family perceptions, and school-level factors can significantly influence constructs such as locus of control, self-efficacy, and resilience. These socioecological variables shape children’s experiences and belief systems, potentially affecting the development and expression of control beliefs and resilient behaviors. Future research should incorporate these constructs to provide a more comprehensive understanding of how environmental factors interact with individual characteristics across different cultural and educational settings.

## 6. Practical Implications

Regardless of the above limitations, the findings of the current study add to the literature exploring the factors that are related to resilience and highlight the importance of integrating locus of control and self-efficacy in intervention programs for the enhancement of children’s resilience. To this end, integrating social-emotional learning (SEL) curricula within school settings may help strengthen students’ self-efficacy and internal locus of control through structured activities focused on emotional regulation, goal-setting, and problem-solving. Increasing children’s internal locus of control and self-efficacy through classroom-based interventions may contribute to improved mental health outcomes. Through these intervention programs, children will learn to attribute their behaviors to internal factors, and this will prevent the manifestation of a range of dysfunctional behaviors or internalizing problems. Taking into account that locus of control is a characteristic that is formed early in development and has a dynamic nature, it is of paramount importance for these programs to be applied when children enter the school and feel the need to balance their interactions with their parents, classmates and teachers (Georgiou et al., 2017).

It is essential to involve the family as well. Intervention programs should not be constrained within the boundaries of the school. Children’s parents should be educated about the impact that their parenting practices have on their children’s development. Therefore, developing parent psychoeducation modules could support the adoption of positive parenting practices that reinforce children’s sense of agency and resilience at home. Ahlin and Lobo Antunes (2015) have shown that a warm and nurturing parental environment plays a significant role in the development of an internal locus of control, whereas harsh discipline is associated with the development of an external locus of control. For this purpose, there is a need for psychoeducation sessions and skills training for parents as well. Towards this direction, pediatric practitioners who have direct communication with families could nurture lifelong resilience by educating/counseling parents about the importance of childhood for building important traits for mental health and well-being. Altogether, these targeted strategies enhance the translational value of the findings and provide concrete guidance for future interventions.

## Figures and Tables

**Table 1:** Means and standard deviations for all variables by gender and grade level.

	Empathy		Goals and Aspirations		Self-Awareness		Community Support		Peer Support		School Support		Home Support		Self-Efficacy		External Locus of Control	
	(Range = 3–12)		(Range = 4–16)		(Range = 6–24)		(Range = 6–24)		(Range = 5–20)		(Range = 5–20)		(Range = 5–20)		(Range = 0–8)		(Range = 0–20)	
Gender/Grade	M	SD	M	SD	M	SD	M	SD	M	SD	M	SD	M	SD	M	SD	M	SD
Gender																		
Boys	9.40	1.996	14.38	1.781	19.55	2.762	20.09	3.859	16.05	3.041	16.61	2.673	18.14	2.116	32.31	4.856	6.51	3.383
Girls	9.81	1.864	14.59	1.758	19.14	2.805	20.12	3.996	16.90	2.611	16.76	2.630	17.82	2.139	31.44	4.655	6.47	3.379
Grade																		
4th	9.62	1.910	14.39	1.823	19.78	2.500	20.37	3.813	16.59	2.949	16.95	2.626	18.12	1.956	32.47	4.382	6.74	3.218
5th	9.57	2.045	14.46	1.795	19.32	2.768	19.76	4.039	16.56	3.015	16.78	2.494	17.88	2.297	31.88	5.379	6.72	3.246
6th	9.66	1.880	14.61	1.658	18.88	3.020	20.10	3.956	16.38	2.605	16.34	2.763	17.88	2.173	31.18	4.555	6.05	3.606
Total	9.62	1.936	14.49	1.758	19.33	2.791	20.11	3.930	16.50	2.849	16.69	2.649	17.98	2.133	31.85	4.766	6.49	3.379

**Table 2:** Measurement invariance across gender for the study measures.

Measures	Model	*CFI*	*RMSEA* (90% CI)	*SRMR*	Model Comparison	Δ*CFI*	Decision
Resilience Youth Development Module							
Internal factors	M1: Configural	0.888	0.052	0.052			
	M2: Metric	0.884	0.051	0.057	2 vs. 1	−0.004	Accept
	M3: Scalar	0.861	0.054	0.057	3 vs. 2	−0.023	Reject
External factors	M1: Configural	0.876	0.060	0.061			
	M2: Metric	0.874	0.059	0.065	2 vs. 1	−0.002	Accept
	M3: Scalar	0.869	0.059	0.064	3 vs. 2	−0.005	Accept
New General Self-Efficacy Scale							
	M1: Configural	0.963	0.062	0.038			
	M2: Metric	0.961	0.059	0.049	2 vs. 1	−0.002	Accept
	M3: Scalar	0.965	0.052	0.045	3 vs. 2	+0.004	Accept
Locus of control scale for children							
	M1: Configural	0.753	0.047	0.057			
	M2: Metric	0.752	0.045	0.061	2 vs. 1	−0.001	Accept
	M3: Scalar	0.702	0.048	0.061	3 vs. 2	−0.050	Reject

Note. *CFI* = comparative fit index; *RMSEA* = root mean square error of approximation; *SRMR* = standardized root mean square residual; Δ*CFI* = change in comparative fit index. Decision based on ΔCFI < 0.0.

**Table 3:** Measurement invariance across grades for the study measures.

Measures	Model	*CFI*	*RMSEA* (90% CI)	*SRMR*	Model Comparison	Δ*CFI*	Decision
Resilience Youth Development Module							
Internal factors	M1: Configural	0.830	0.067	0.065			
	M2: Metric	0.812	0.067	0.073	2 vs. 1	−0.018	Reject
	M3: Scalar	0.807	0.065	0.071	3 vs. 1	−0.023	Reject
External factors	M1: Configural	0.858	0.065	0.070			
	M2: Metric	0.855	0.064	0.076	2 vs. 1	−0.003	Accept
	M3: Scalar	0.843	0.064	0.075	3 vs. 2	−0.012	Reject
New General Self-Efficacy Scale							
	M1: Configural	0.946	0.075	0.046			
	M2: Metric	0.949	0.065	0.055	2 vs. 1	−0.003	Accept
	M3: Scalar	0.944	0.063	0.054	3 vs. 2	−0.005	Accept
Locus of control scale for children							
	M1: Configural	0.732	0.050	0.065			
	M2: Metric	0.734	0.048	0.069	2 vs. 1	+0.002	Accept
	M3: Scalar	0.696	0.049	0.070	3 vs. 2	−0.038	Reject

Note. *CFI* = comparative fit index; *RMSEA* = root mean square error of approximation; *SRMR* = standardized root mean square residual; Δ*CFI* = change in comparative fit index. Decision based on Δ*CFI* < 0.01.

**Table 4:** Correlation matrix.

	1	2	3	4	5	6	7	8	9
1. Empathy	-								
2. Goals and aspirations	0.280 **	−							
3. Self-awareness	0.407 **	0.284 **	−						
4. Community support	0.279 **	0.212 **	0.297 **	−					
5. Peer support	0.326 **	0.275 **	0.353 **	0.339 **	−				
6. School support	0.293 **	0.234 **	0.396 **	0.379 **	0.322 **	−			
7. Home support	0.272 **	0.287 **	0.324 **	0.380 **	0.344 **	0.449 **	−		
8. Self-efficacy	0.249 **	0.328 **	0.496 **	0.218 **	0.261 **	0.326 **	0.297 **	−	
9. External locus of control	−0.182 **	−0.179 **	−0.229 **	−0.170 **	−0.140 **	−0.171 **	−0.234 **	−0.196 **	-

** *p* < 0.01.

**Table 5:** Multiple regression analysis predicting resilience.

	Empathy		Goals and Aspirations		Self-Awareness		Community Support		Peer Support		School Support		Home Support	
Predictors	Step (ΔR^2^)	β (Final)	ΔR^2^	β (Final)	ΔR^2^	β (Final)	ΔR^2^	β (Final)	ΔR^2^	β (Final)	ΔR^2^	β (Final)	ΔR^2^	β (Final)
Gender	1 (0.01)	0.12 ***							1 (0.02)	0.17 ***	1 (0.10)	−0.07 *	1 (0.01)	−0.05
Grade					1 (0.02)	−0.10 *								
Self-efficacy	2 (0.07)	0.23 ***	1 (0.11)	0.30 ***	2 (0.23)	0.45 ***	1 (0.05)	0.19 ***	2 (0.08)	0.26 ***	2 (0.10)	0.30 ***	2 (0.08)	0.25 ***
External locus of control	3 (0.02)	−0.13 ***	2 (0.01)	−0.12 ***	3 (0.02)	−0.15 ***	2 (0.02)	−0.13 ***	3 (0.01)	−0.09 *	3 (0.01)	−0.12 **	3 (0.03)	−0.18 ***
R^2^	0.10		0.12		0.27		0.06		0.10		0.12		0.12	

* *p* < 0.05, ** *p* < 0.01, *** *p* < 0.001.

## Data Availability

Data is available upon request from the corresponding author.
